# *Theragra chalcogramma* Hydrolysates, Rich in Gly-Leu-Pro-Ser-Tyr-Thr, Exerts Anti-Photoaging Potential via Targeting MAPK and NF-κB Pathways in SD Rats

**DOI:** 10.3390/md20050286

**Published:** 2022-04-24

**Authors:** Defeng Xu, Mouming Zhao, Haisheng Lin, Caihong Li

**Affiliations:** 1 Guangdong Provincial Engineering Technology Research Center of Marine Food, Guangdong Provincial Key Laboratory of Aquatic Product Processing and Safety, College of Food Science and Technology, Guangdong Ocean University, Zhanjiang 524088, China; xudefeng@gdou.edu.cn; 2 Collaborative Innovation Center of Seafood Deep Processing, Dalian Polytechnic University, Dalian 116034, China; 3 School of Food Science and Engineering, South China University of Technology, Guangzhou 510640, China; femmzhao@scut.edu.cn; 4 School of Basic Medical Sciences, Guangdong Medical University, Dongguan 523808, China

**Keywords:** skin photoaging, *Theragra chalcogramma*, oxidative stress, inflammation, cascading signaling

## Abstract

Previous studies have revealed that excessive exposure to UV irradiation is the main cause of skin photoaging and the signaling pathways of MAPK and NF-κB are involved in this progression. The present study aims to investigate the anti-photoaging effects of low molecular weight hydrolysates from *Theragra chalcogramma* (TCH) and to clarify the underlying mechanism. The degradation of mechanical barrier functions in photoaged skin was substantially ameliorated after TCH administration; meanwhile, TCH significantly elevated the antioxidant capacity and suppressed the over-production of inflammatory cytokine IL-1β. Moreover, the histopathological deteriorations such as epidermal hyperplasia and dermal loss were significantly alleviated, along with the increase in procollagen type I content and decrease in MMP-1 activity (*p* < 0.05). Furthermore, TCH effectively blocked the MAPK and NF-κB signaling pathways through inhibition of the phosphorylation of p38, JNK, ERK, iκB, and p65 proteins. Collectively, these data indicate that TCH has potential as a novel ingredient for the development of anti-photoaging foods.

## 1. Introduction

Skin is susceptible to environmental fluctuations and solar ultraviolet (UV) radiation constitutes the most noxious factor inducing skin damage. Biochemically, excessive UV exposure induces oxidative stress and eventually leads to photoaging in cutaneous tissue, which is characterized by deep and coarse wrinkles, solar scars, roughness, dryness, laxity, and pigmentation [1,2,3]. Generally, photoaging is a progressive and complex process during which UV radiation stimulates the excessive release of reactive oxygen species (ROS) and induces the epidermal hyperplasia, collagen degradation, and disintegration of elastic fibers [4,5,6]. During the past several decades, considerable reports have revealed the involvement of ROS in the damage of biological macromolecules such as DNA, lipids, and proteins, and in the decrease in antioxidant enzyme activities such as superoxide dismutase (SOD), catalase (CAT), and glutathione peroxidase (GSH-Px) [7,8,9]. Moreover, excessive ROS activates the mitogen-activated protein kinase (MAPK) signaling pathway and subsequently leads to activation of the nuclear transcription factor, with the consequences of a dramatic release of inflammatory cytokines and an abnormal activation of nuclear factor kappa B (NF-κB) signaling pathway [1,2,4]. Eventually, the expression of matrix metalloproteinases (MMPs) is immediately stimulated and the degradation of extracellular matrix (ECM) in the skin’s dermal layer is accelerated. In view of the roles of oxidative stress and inflammation in the process of photoaging, it is therefore reasonable to develop functional ingredients with antioxidative and anti-inflammatory capacity to prevent photoaging.

Consequently, obtaining antioxidative and anti-inflammatory ingredients such as polysaccharides, polyphenols, and protein hydrolysates from natural dietary sources has attracted increasing attention in recent years [10,11,12,13,14]. To date, the excellent performance in scavenging activity of free radicals of some typical edible protein hydrolysates such as corn gluten meal with dairy whey [15], milk protein hydrolysates [16], lotus (*Nelumbo nucifera Gaertn*). seed protein hydrolysates [17], and defatted walnut meal hydrolysate [18] has been demonstrated and these used as antioxidants in the food industry. Notably, an upsurging interest in the utilization of food protein hydrolysates has been witnessed in the past few decades for their excellent antioxidant and anti-inflammatory performances. Moreover, it is generally accepted that the bioactivities of proteins and hydrolysates result from the special fragments of amino acid residues. As for the photoaging intervention, many food-derived antioxidant hydrolysates have exerted desirable interventions on photoaging progression, and, in particular, collagen and its hydrolysates have been increasingly developed as food additives, cosmetics, biomedical materials, and pharmaceuticals because of their excellent biocompatibility, biodegradability, and low antigenicity [19,20,21,22,23].

In addition, collagen and elastin are the principal components in ECM and play vital roles in providing skin with tensile strength and elasticity. During photoaging progression, the reduction in collagen and elastin fibers causes the local collapse in the dermal layer, and dietary supplement of protein hydrolysates, therefore, is a useful strategy to stimulate the expression of collagen and elastin for alleviating photoaging. In recent decades, numerous hydrolysates from collagen resources have demonstrated their excellent bioactivities, whereas little attention has been paid to the hydrolysates from marine elastin for photoaging alleviation. As a commercially important fish species in China and throughout the world, the biomass of by-products processed from *Theragra chalcogramma* is increasing, but studies in the literature have only reported about the peptides release from *Theragra chalcogramma* so far. Using hydroxyapatite affinity chromatography, Jung et al. (2006) obtained low molecular weight peptides from the discarded backbone protein of *T. chalcogramma* processing and revealed its high affinity to calcium [24]. Yang et al. (2018) prepared the low molecular weight peptides from Alaska Pollock with enzymatic hydrolysis and demonstrated their excellent wound healing capacity [25]. However, no literature on the use of hydrolysates from *T. chalcogramma* against photoaging is available. Therefore, the present study aims to: (1) prepare the low molecular weight hydrolysates from *T. chalcogramma* with potent anti-photoaging performance, and (2) to clarify the underlying mechanism. For this purpose, the capacity of the antioxidant and antiinflammatory response, the deposition of ECM components, and the inactivation of the MAPK and NF-κB pathways are systematically examined. Altogether, this study not only gives an in-depth understanding of TCH against photoaging, but also sets a solid foundation for the development of TCH as a functional agent.

## 2. Results and Discussion

### 2.1. TCH Improved the Appearance and Barrier Functions of Photoaged Skin

**Figure 1:**
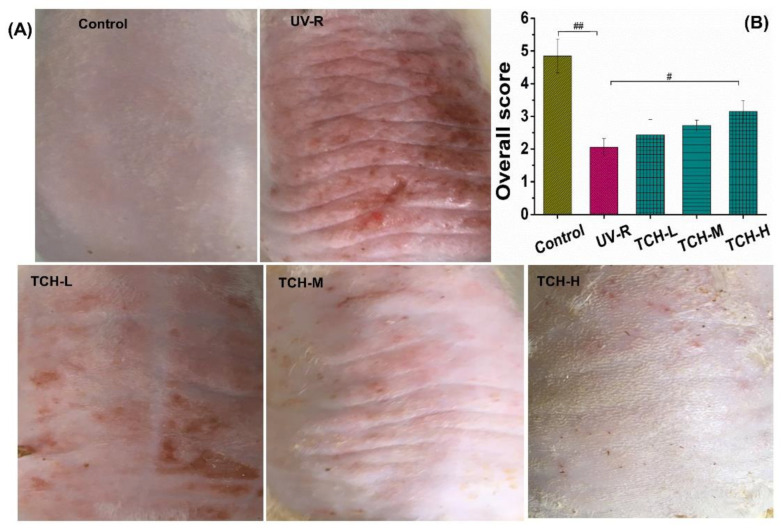
Effects of TCH intervention on the (**A**) macro-observations, and (**B**) overall score of photoaging SD rat. # and ## indicate significant difference at *p* < 0.05 and *p* < 0.01 level, respectively, when compared with that in UV-R group.

Deep wrinkles and a coarse appearance are typical features of the morphology of photoaged skin. As shown in Figure 1A,B, the dorsal skin of SD rats in the normal group was smooth and few shallow wrinkles were observed; meanwhile, the overall score was as high as 4.55 ± 0.52. In the UV-R group, however, the macro-observations substantially degraded, along with the deep wrinkles, leathery appearance, and a significant decrease in the visual score (*p* < 0.01). After oral administration of TCH, the gradual alleviation of the deep wrinkles was observed and the overall score was significantly elevated in the TCH-H group (*p* < 0.05). 

**Figure 2:**
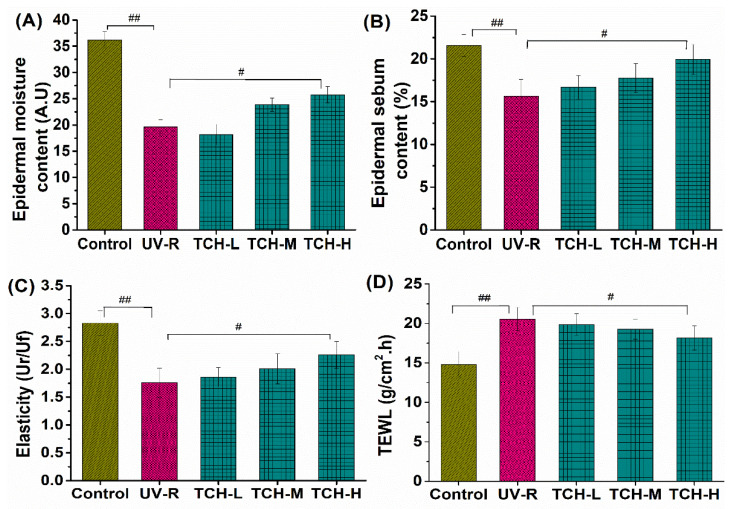
Effects of TCH intervention on the mechanical barrier functions of photoaged skin. (**A**) Change in epidermal moisture content; (**B**) change in epidermal sebum content; (**C**) change in skin elasticity; (**D**) change in TEWL. # and ## indicate significant difference at *p* < 0.05 and *p* < 0.01 level, respectively, when compared with that in UV-R group.

In addition, the values of epidermal moisture, sebum contents, and elasticity in the normal group were 35.25 ± 1.27 A.U. (Amplitude Unit), 20.42 ± 1.69%, and 2.54 ± 0.26 Ur/Uf (the ratio of elastic recovery to the total deformation), respectively. However, these corresponding values significantly decreased in the UV-R group (all *p* < 0.01, Figure 2). Conversely, transepidermal water loss (TEWL) significantly increased from 15.24 ± 0.69 g/cm^2^·h in the normal control group to 22.41 ± 0.72 g/cm^2^·h in the UV-R group (*p* < 0.01), whereas a gradual reduction in TEWL was observed after TCH administration. These findings revealed the excellent amelioration role of TCH on mechanical barrier functions in photoaged skin.

### 2.2. TCH Alleviated the Pathological Impairments of Photoaged Skin

**Figure 3:**
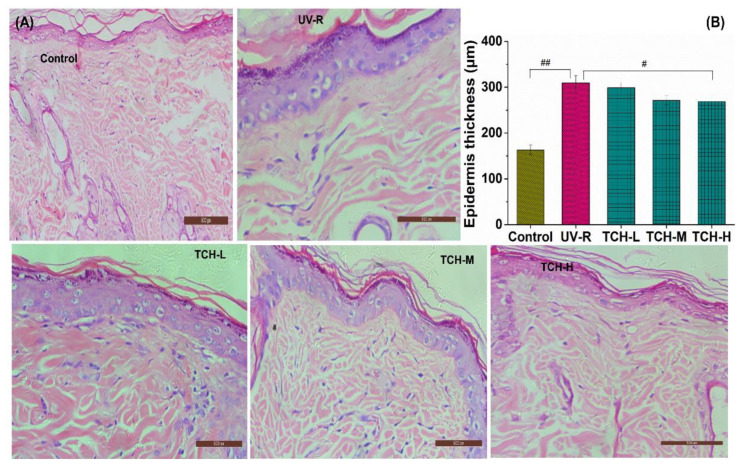
Effects of TCH on the histopathological abnormalities of epidermal sections in photoaged skin. (**A**) Representative photographs of sections by hematoxylin and eosin staining (200× magnification); (**B**) change in epidermal thickness. # and ## indicate significant difference at *p* < 0.05 and *p* < 0.01 level, respectively, when compared with that in UV-R group.

As shown in Figure 3A, the orderly arrangement of keratinocytes and fibroblasts and few degenerations of collagen fibers were observed in the normal control group. However, both epidermal hyperplasia and a loss of fibroblasts appeared in the UV-R group, indicating the obvious pathological impairments under chronic UV irradiation. With the administration of TCH, this epidermal hyperplasia was gradually alleviated. As illustrated in Figure 3B, the epidermal thickness in the normal control group was 155.24 ± 13.22 μm and the corresponding value in the UV-R group was significantly increased to 302.38 ± 14.57 μm, significantly higher than that in the normal control group (*p* < 0.01). With the administration of TCH, the epidermal thickness in the TCH-H group significantly decreased to 228.16 ± 13.27 μm (*p* < 0.05), suggesting the substantial alleviation of epidermal hyperplasia. 

**Figure 4:**
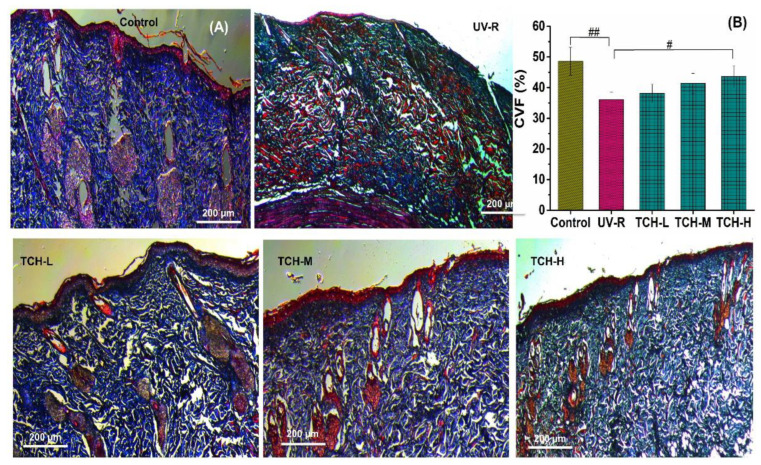
Effects of TCH on the histopathological abnormalities of dermis sections in photoaged skin. (**A**) Representative photographs of sections by Masson staining (200× magnification); (**B**) change in CVF. # and ## indicate significant difference at *p* < 0.05 and *p* < 0.01 level, respectively, when compared with that in UV-R group.

Furthermore, the collagen fibril in the dermal layers can be stained with the Masson trichrome method, and the abundant production and good organization of collagen are characterized by the thinner and more densely organized collagen bundles [11]. In the present study, the waving arrangement and deep blue color were illustrated in the normal group (Figure 4A). In the UV-R group, however, the light blue of collagen fibers was observed, which indicated the substantial reduction in collagen density. With TCH administration, the deposition of collagen fibril was effectively elevated and the damaged architecture was gradually restored in a dose-dependent manner, indicating the powerful repairing efficacy of TCH on the destroyed dermal structure. Moreover, the collagen volume fraction (CVF) in the normal group was 52.33 ± 1.36%, whereas the corresponding value significantly dropped to 34.17 ± 1.59% in the UV-R group. After intervention with TCH, the value increased in a dose-dependent manner and the significant enhancement of 44.38 ± 1.69% was observed in the TCH-H group (*p* < 0.05, Figure 4B). This result reveals the impairment of chronic UV irradiation on the skin’s architecture and the substantial restoration provided by TCH on the biosynthesis of collagen fibers. The phenotypes of skin photoaging are the consequence of a net deficit of connective tissue and the wrinkles’ formation mainly results from the ECM degradation [26,27,28]. Therefore, the attenuation in epidermal hyperplasia and dermal loss revealed the powerful restoration ability of TCH on the damaged architecture of the ECM in photoaged skin.

### 2.3. TCH Elevated the Antioxidative Capacity and Suppressed the Inflammation in Photoaged Skin

**Figure 5:**
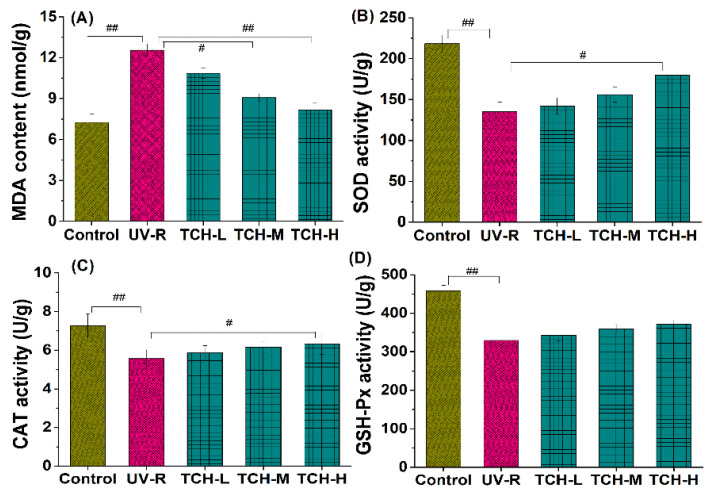
Effects of TCH on the MDA levels and activities of antioxidant enzymes in photoaged skin. (**A**) Change in MDA level; (**B**) change in CAT activity; (**C**) change in SOD activity; (**D**) change in GSH-Px activity. # and ## indicate significant difference at *p* < 0.05 and *p* < 0.01 level, respectively, when compared with that in UV-R group.

It is well known that an excessive accumulation of ROS leads to the abnormal production of MDA and substantially depletes the antioxidant capacity. As illustrated in Figure 5A, a significant increase in MDA content was observed in the UV-R group (*p* < 0.01), indicating the high level of oxidative stress. With the intervention of TCH, MDA content gradually decreased and a significant reduction was observed in the TCH-H group (*p* < 0.01). In addition, all the activities of SOD, CAT, and GSH-Px in UV-R group significantly decreased in comparison with those in the normal control group (all *p* < 0.01, Figure 5B–D). With TCH administration, the activities of SOD, CAT, and GSH-Px were enhanced in a dose-dependent manner and the significant enhancement of CAT and SOD was observed in the TCH-H group (*p* < 0.05). These findings indicate the dramatic attenuation effect of TCH on oxidative stress and the significant elevation in antioxidant capacity. 

**Figure 6:**
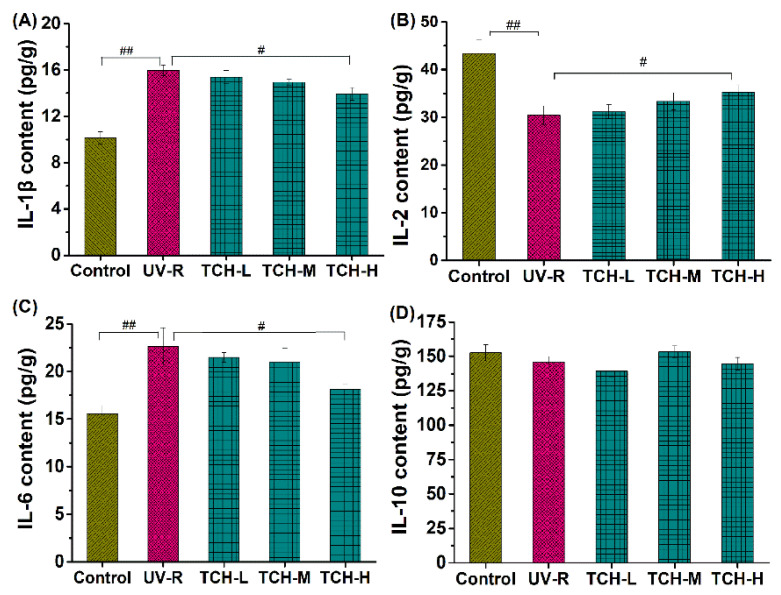
Effect of TCH on the inflammatory cytokines in photoaged skin of SD rats. (**A**) Change in the level of IL-1β; (**B**) change in the level of IL-6; (**C**) change in the level of IL-2; (**D**) change in the level of IL-10. # and ## indicate significant difference at *p* < 0.05 and *p* < 0.01 level, respectively, when compared with that in UV-R group.

Moreover, numerous investigations have revealed that photoaging disrupts the homeostasis of inflammatory cytokines, and the intervention on inflammatory cytokine production is therefore beneficial to alleviate photoaging. In the present study, photoaging significantly promoted the release of inflammatory cytokines IL-1β and IL-6, whereas it downregulated the production of anti-inflammatory cytokines IL-2 (*p* < 0.01, Figure 6A,B), indicating the occurrence of significant inflammation. With the administration of TCH, the above imbalance in inflammatory and anti-inflammatory cytokines was gradually alleviated and a significant modulation was observed in the TCH-H group (*p* < 0.05). This finding demonstrates the desirable modulation capability of TCH on the imbalance of cytokines in photoaged skin. The modulation of food-derived peptides on cytokine imbalance has been reported by several studies [20,21,25,29], and our finding is consistent with these reports.

**Figure 7:**
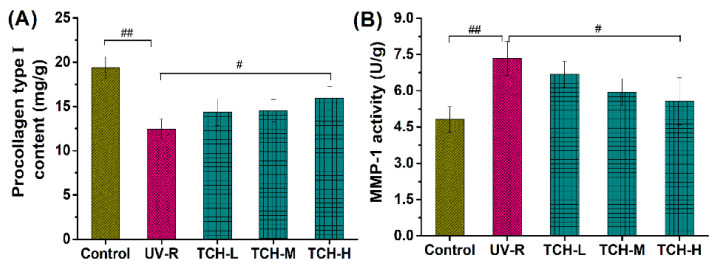
Effects of TCH on the (**A**) contents of procollagen type I, and (**B**) MMP-1 activity in photoaged skin. # and ## indicate significant difference at *p* < 0.05 and *p* < 0.01 level, respectively, when compared with that in UV-R group.

Furthermore, photodamage is closely associated with the reduction in procollagen type I and the significant elevation of MMPs, during which oxidative stress and the inflammation response mediate the cascading activation of MMPs’ expression and lead to the degradation of ECM components in the dermal layer [10,12,14,30,31,32]. Herein, the contents of procollagen type I in the control group significantly decreased, while the MMP-1 activity significantly increased after chronic UV irradiation for 18 weeks (Figure 7, both *p* < 0.01), indicating the dramatic reduction in the collagen components in the dermal layer. With the administration of TCH, both the reduction in procollagen type I and the increase in MMP-1 activity were reversed in a dose-dependent manner, among which a significant reversion was observed in the TCH-H groups (*p* < 0.05), demonstrating the potent stimulation of TCH on collagen biosynthesis.

### 2.4. TCH Attenuated the Hyperactivation of MAPK and NF-κB Pathways in Photoaged Skin

**Figure 8:**
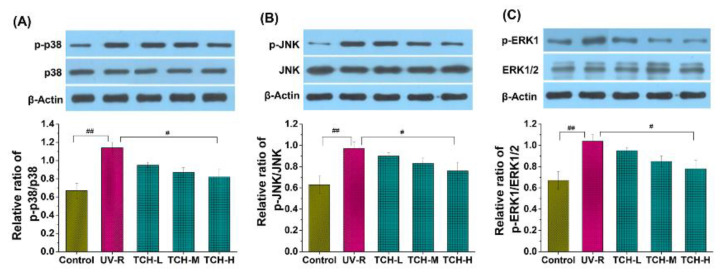
Effect of TCH on the activation of MAPK signaling pathway in photoaged skin. (**A**) Change in the phosphorylation level of p38; (**B**) change in the phosphorylation level of JNK; (**C**) change in the phosphorylation level of ERK. # and ## indicate significant difference at *p* < 0.05 and *p* < 0.01 level, respectively, when compared with that in UV-R group.

Generally, an excessive accumulation of ROS induces activation of the MAPK pathway, in which the phosphorylation levels of principal kinases such as ERK, JNK, and p38 dramatically increase [3,7,10,14]. As shown in Figure 8, the phosphorylation levels of ERK, JNK, and p38 in the UV-R group were significantly elevated as compared to those in the normal control group, which indicated the substantial activation of the MAPK signaling pathway. With TCH administration, the phosphorylation levels of ERK, JNK, and p38 proteins were gradually reduced and a significant reduction was observed in the TCH-M (p38 and ERK, both *p* < 0.05) and TCH-H groups (p38, JNK and ERK, all *p* < 0.01), demonstrating the excellent inhibition activity of TCH on the MAPK signaling pathway. Additionally, numerous studies in the literature have revealed that the MAPK signal pathway mediated the upregulation of MMPs [1,7,30,31]. As a result, in view of the cascading association of MAPK and MMPs, we have reason to postulate that TCH might ameliorate photoaging by inactivating the hyperphosphorylation of the JNK, ERK, and p38 proteins and, subsequently, suppressing the MMP-1 activity; but the actual situations need to be further explored by using MAPK inhibitors. 

**Figure 9:**
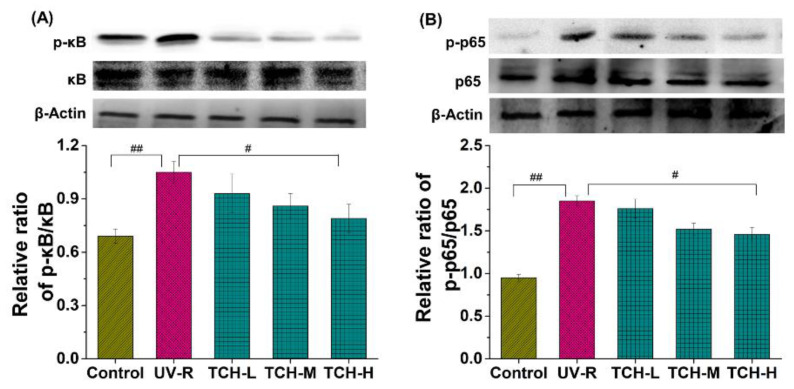
Effect of TCH on the activation of NF-κB signaling pathway in photoaged skin. (**A**) Change in the phosphorylation of κB; (**B**) change in the phosphorylation of p65. # and ## indicate significant difference at *p* < 0.05 and *p* < 0.01 level, respectively, when compared with that in UV-R group.

In addition, Figure 9 illustrates the significant elevation of the phosphorylation levels of κB and p65 proteins in the UV-R group as compared to those in the normal group (*p* < 0.05 and *p* < 0.01, respectively), whereas no obvious difference was observed in the κB and p65 band intensity between the normal and UV-R groups. These data indicated the activation of the NF-κB signaling pathway. With the intervention of TCH, the band intensity of p-κB and p-p65 reduced in a dose-dependent manner and a significant reduction was observed in the TCH-H group (both *p* < 0.05). This finding suggested the prominent inhibition activity of TCH on the activation of the NF-κB signaling pathway by blocking phosphorylation of the κB and p65 proteins. Many reports have demonstrated that ROS triggers the NF-kB activation by elevating the phosphorylation levels of κB and p65 proteins, following with the translocation of NF-κB into the nucleus and the stimulation of MMPs’ expression [1,4,12,27,32,33]. The anti-photoaging effect of several protein hydrolysates resulted from their inhibition of MMP-1 activity [34], and these reports are consistent with our finding.

Generally, it is believed that the amino acid composition plays a key role in the bioactivity of peptides, and the action mechanism of bioactive peptides mainly results from providing hydrogen atoms or electrons to participate in scavenging free radical reactions. The antioxidant peptides usually contain a hydrophobic amino acid residue at the N-terminal, which acts as a hydrogen donor and enhances the antioxidant capacity. Sheng et al. (2019) prepared the protein hydrolysate from defatted walnut meal hydrolysate, identified the antioxidant peptides fragments DWMPH, and explored the antioxidative mechanism from the perspective of structure-based screening [18]. Their findings demonstrated that two mechanisms of hydroxyl radical scavenging and ROS reduction were involved in their antioxidative effects at different degrees. In the present study, the peptide fragment Gly-Leu-Pro-Ser-Tyr-Thr had the abundant hydrophobic amino acid residue of Leu, Pro and Tyr. Therefore, TCH exhibited powerful hydroxyl radical scavenging and ROS reduction activities and this might be the action mechanism against photoaging. 

**Figure 10:**
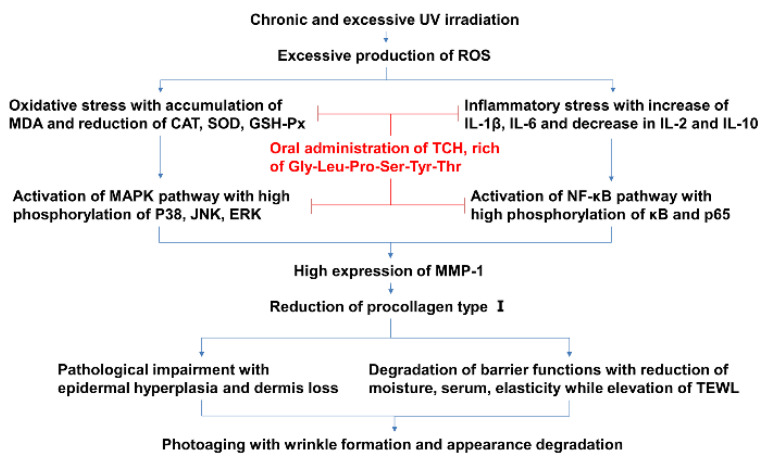
The potential cascading mechanism of TCH against photoaging.

Skin photoaging is induced by chronic and excessive UV irradiation and is characterized by epidermal hyperproliferation, coarse wrinkles, and rough appearance. Considerable reports have revealed the intervention effect of antioxidative and anti-inflammatory agents such as the food-derived protein hydrolysates on photoaging progression [1,10,23,29,34,35]. Therefore, based on these close cascading associations, we speculate on the potential mechanism of TCH against photoaging. As shown in Figure 10, chronic and excessive UV irradiation initially induces severe oxidative stress, which subsequently leads to imbalance of the inflammatory cytokines, and, consequently, activates the MAPK and NF-κB signaling pathways, followed with the reduction of collagen biosynthesis, impairment of architecture, degradation of barrier functions, and, eventually, the appearance of typical photoaging morphology. With the administration of TCH, photoaging progression is significantly suppressed by synergistic modulation through the elevation of antioxidant capacity, suppression of inflammatory cytokines, inactivation of the MAPK and NF-κB signaling pathways, and the stimulation of collagen biosynthesis. This investigation deepens the understanding of the effect of food-derived peptides against photoaging and sets a solid foundation for the development of TCH as a functional agent. 

## 3. Materials and Methods

### 3.1. Preparation of TCH and Identification of Main Components with Antioxidant Activity

**Figure 11:**
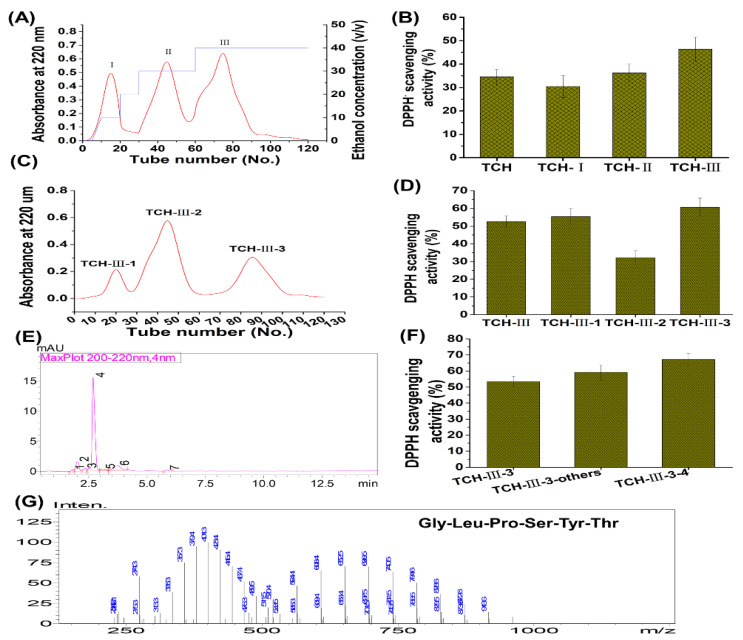
Preparation, isolation, and characterization of TCH with high antioxidant capacity. The low molecular weight hydrolysates in TCH were firstly isolated with SP-825 macroporous adsorption resin (**A**) and the DPPH radical scavenging activity of TCH and its three fractions were compared (**B**); then the fraction TCH-III with highest DPPH radical scavenging activity was subsequently isolated with Sephadex G-15 column (**C**) and the fraction TCH-III-3 showed the highest scavenging activity on DPPH radical among the three subfractions (**D**); next, fraction TCH-III-3 was further isolated with RP-HPLC (**E**) and TCH-III-3-3 exhibited the highest scavenging activity (**F**); eventually, the fraction TCH-III-3-3 was analyzed with LC-MS/MS and the principal fragments were identified accordingly (**G**).

Low molecular weight TCH was prepared by enzymatic hydrolysis as described by Yang et al. (2018) [25] and Liu et al. (2018) [29]. Briefly, the fresh fish *T. chalcogramma,* with average weight of 521.65 ± 21.38 g, were purchased from a local supermarket in Zhanjiang City, China. The skin was collected and autoclaved to liquefy at high temperature and pressure. Then the extract was hydrolyzed with thermolysin at the ratio of enzyme to substrate (E/S) of 0.3% (*m*/*v*), 48 °C, and pH 7.0 for 5 h. After incubation, hydrolysis was quenched by heating and the hydrolysates were centrifuged at 12,000× *g* and 4 °C for 15 min, followed with filtration of supernatant through 3 kDa MWCO ultrafiltration membrane (Millipore, NJ, USA) to acquire the short-chain peptides (<3 kDa). Furthermore, the isolation and identification of TCH were conducted with macroporous adsorption resin, gel permeation chromatography, and medium pressure liquid chromatography preparation system as described by Kim et al. (2018) [20]. The radical scavenging capacity of TCH on 1,1-diphenyl-2-picryl-hydrazyl (DPPH (Sigma, D21140-0, St. Louis, MO, USA)) was used to evaluate the antioxidant activity of each fraction. Briefly, 0.5 mL of isolation solution was mixed with 3.5 mL of DPPH solution (0.1 mmol/L). After shaking for 15 s, the mixed solution was kept in the dark for 30 min and then measured at 520 nm. The aqueous solution was used as a blank solution and DPPH aqueous solution was used as a control solution. All experiments were performed in triplicate. The DPPH radical scavenging capacity of fractions was expressed in terms of % inhibition. As shown in Figure 11, among TCH and its three fractions (Figure 11A), fraction TCH-III exhibited the highest DPPH hydroxyl radical scavenging activity (Figure 11B) and was then isolated with Sephadex G-15 column (Figure 11C). Among the isolated subfractions, TCH-III-2 showed the highest radical scavenging activity (Figure 11D) and was further isolated with RP-HPLC (Figure 11E). Overall, TCH-III-2-2 exhibited a higher scavenging activity than other subfractions (Figure 11F) and, eventually, the principal fragment Gly-Leu-Pro-Ser-Tyr-Thr was identified with UPLC-ESI-MS/MS (Bruker Daltonics Inc., Billerica, MA, USA) in the positive electrospray ionization (ESI^+^) mode via the electrospray interface. Spectra were recorded over the mass/charge (*m*/*z*) range 300–1400 and the peptide sequencing was performed through processing the auto multiple MS (Auto MS/MS) spectra program. 

### 3.2. Materials and Chemicals 

The depilatory sodium sulfide was purchased from Hong Ming Chemical Reagent Co., Ltd. (Jining, China). Commercial kits for detection of MDA, SOD, CAT, GSH-Px, procollagen type I, total MMP-1, hematoxylin–eosin (H&E) and Masson staining were from Jiancheng Institute of Biotechnology (Nanjing, China). The commercial kits IL-1β, IL-2, IL-6, IL-10 were purchased from eBioscience, Inc. (San Diego, CA, USA). All other chemicals and reagents used in present study were of analytical grade. The primary and corresponding secondary antibodies of β-actin, p-ERK/ERK, p-JNK/JNK, p-p38/p38, p-IκB/IκB, and p-p65/p65 were purchased from Santa Cruz Biotechnology (Santa Cruz, CA, USA). Tris-buffered saline with 0.1% Tween 20 (TBST) was purchased from Merck (Merck & Co., Inc., Kenilworth, NJ, USA). Animal feeds and shaving pad were purchased from Xinhua Experimental Animal Factory (Huadu District, Guangzhou, China). 

### 3.3. Rats and Grouping

Sixty healthy adult female SD rats 180 ± 10.15 g were purchased from the experimental animal center in Guangzhou University of Chinese Medicine. The experiments were approved by the Animal Ethics Committee of Guangdong Ocean University with the approval number of SYXK 2019-0053, approved on 28 April 2019. After acclimation in an airconditioned room (25 ± 2 °C, 65–70% humidity) and free access to tap water for one week, the rats were randomly assigned into 5 groups (n = 12): (1) normal control group, in which the rats were not exposed to UV irradiation and had free access to tap water; (2) photoaging model group, UV-R, in which the rats were exposed to UV irradiation and had free access to tap water; (3) TCH group at low dosage, TCH-L, in which the rats were exposed to UV irradiation and had free access to tap water containing 0.32 g/100 mL of TCH; (4) TCH group at medium dosage, TCH-M, in which the rats were exposed to UV irradiation and had free access to tap water containing 0.96 g/100 mL of TCH; (5) TCH group at high dosage, TCH-H, in which the rats were exposed to UV irradiation and had free access to tap water containing 2.88 g/100 mL of TCH.

### 3.4. Establishment of Photoaging Model

UV irradiation was carried out according to our previous report [36]. Briefly, apart from the SD rats in control group, other animals were fixed on a self-made panel and exposed to the irradiation apparatus, which was installed with the UVA (Philips 340-40WT12-G13), UVB (Philips 313-40WT12-G13 lamps), and corresponding radiometer (Beijing Normal University of Photoelectric Instrument Factory, Beijing, China) for UVA and UVB, respectively. The distance from lamps to rats was set at 40 cm and the irradiation intensity was monitored by radiometer. UV-R was conducted one time in every two day’s interval and the time of each irradiation lasted for 15 min in first week, 20 min in second week, 30 min in third week up to the end of experiment, respectively. At the end of radiation, intensity of UV-A and UV-B was accumulated to 150.88 and 78.67 mW/cm^2^, respectively. After consecutive UV radiation for 18 weeks, the obvious photoaging morphology in dorsal skin of UV-R group was observed and this observation indicated the establishment of photoaging animal model.

### 3.5. Evaluation of Appearance and Barrier Functions

The overall appearance and indicators of epidermal moisture and sebum content, skin elasticity, and TEWL were measured subsequently according to our previous report [36]. Briefly, the dorsal skin was cleaned with distilled water and then the wrinkle scale in dorsal skin was evaluated as described by Inomata et al. (2003) [37]. For evaluation of overall score, the skins in normal control group had almost no wrinkles observed and the overall score was set as 5, while the skins with a few shallow wrinkles were scored 4. Next, the skins with several shallow wrinkles were scored 3 and those with some deep and long wrinkles were scored 2. Finally, the skin with many deep and long wrinkles were set as 1. The epidermal moisture and sebum content, and elasticity in depilated area were measured using FC1502 Facecaie Skin Analyzer (Shenzhen Kier electronic apparatus factory, Shenzhen, China) according to the instrument’s specification. TEWL was measured quantitatively using Tewameter (TM300, Courage + Khazaka, Cologne, Germany) and the value was automatically expressed as g/m^2^ h. 

### 3.6. Histological Observation

The skin tissues were embedded in paraffin and 6 μm of sections were cut for hematoxylin and eosin (H&E) staining according to Takeuchi et al. (2010) [38]. The collagen deposition in dermis was stained with Masson’s trichrome staining procedure described by Yu et al. (2016) [39]. Images were visualized by a light microscope (Olympus BX51, Olympus Co., Tokyo, Japan) and digital imaging system (Olympus DP71, Olympus Co.). The collagen volume fraction (CVF) was calculated as the ratio of collagen area to total area. 

### 3.7. Determination of Biochemical Indicators in Skin Tissue

At the end of final UV irradiation, the rats were anesthetized with diethyl ether and sacrificed. Then the typical dorsal skin was sampled and 10% tissue homogenate was prepared according to the routine protocol. The content of MDA, IL-1β, IL-2, IL-6 and IL-10, and procollagen type I, and the activities of CAT, SOD, GSH-Px, and MMP-1 were subsequently detected with commercial kits from Nanjing Jiancheng Bioengineering Institute (Nanjing, Jiangsu, China) according to the manufacturer’s corresponding protocols. The activity of CAT, SOD, GSH-Px was detected based on the inhibitory effect on generation rate of superoxide anion by xanthine and xanthime oxidase reaction system. One unit of SOD was defined as the activity amount that resulted in 50% inhibition of the production of superoxide anion in 200 μL of reaction solution, and the results were expressed as specific activity in the unit of U/g skin tissue. One unit of CAT catalytic activity was defined as the amount of enzyme required to decompose of 1 μmol hydrogen peroxide per second and the enzyme activity was expressed as U/g skin tissue. 

### 3.8. Assay of MAPK and NF-κB Signaling Pathway

The expression levels of total and phosphorylation of p38, JNK, ERK, κB, and p65 proteins in skin tissue were detected according to Kim et al. (2018) [20] and Verma et al. (2017) [30] with slight modifications. Briefly, after separated by 12% SDS-PAGE, the protein was transferred to nitrocellulose membranes and blocked with bovine serum albumin, followed with incubation in the corresponding primary antibodies or β-actin solution at 4 °C overnight. Next, by washing with TBS-Tween and incubating with the appropriate secondary antibody, the proteins were detected with enhanced chemiluminescent reagent and the densitometry of bands was quantified using Image-Pro Plus 6.0 system (Media Cybernetics, Georgia Avenue, Silver Spring, MD 20910 USA). 

### 3.9. Statistical Analysis

All data were expressed as means ± standard deviation of three independent determinations. Statistical comparisons between different groups were performed using one-way analysis of variance (ANOVA). # and ## indicate significant difference at *p* < 0.05 and *p* < 0.01 level, respectively, when compared with that in UV-R group.

## 4. Conclusions

Low molecular weight hydrolysate from *T. chalcogramma* exhibited potent amelioration of photoaging, and the underlying mechanism of TCH against photoaging progression might lie in the synergistic modulation of the hyperactivated MAPK and NF-κB signaling pathways by reversing the imbalance of oxidative and inflammatory stress, stimulation of collagen deposition, restoration of histopathological impairments, and eventual elevation of barrier functions.

## Data Availability

Not applicable.
